# Meals and Movies: What Makes Our Microbiota Merry?

**DOI:** 10.1192/j.eurpsy.2023.2035

**Published:** 2023-07-19

**Authors:** O. Yousef, S. De Souza

**Affiliations:** 1 University of Bristol, Bristol; 2 Somerset NHS Foundation Trust, Somerset, United Kingdom

## Abstract

**Introduction:**

A healthy microbiota should be on all our Christmas lists this year. There is compelling evidence that good gut health is associated with better mental health, especially important during these cold winters. To spark some joy during this time, many of us enjoy a festive film and we can probably admit we also overindulge during the festive season.

**Objectives:**

We aim to investigate “what is the impact of festive cinematic diets on the gut microbiota?”.

**Methods:**

We identified films and broke down the festive meals into their constituents. Using our MINCE PIE (Microbiota INdex of Comparative Evaluation for Pictorial Infographic Evidence) scoring equation (=Microbiota Enhancing Food Groups - Microbiota Detrimental Food Groups), we formulated scores for 12 festive films. We sought to rate meals in each film to assess their relative ability to enhance the gut microbiotia.

**Results:**

Most festive films contained meals or foods from a typical “Western diet” i.e., high sugar/high fat. These meals overall show negative effects. However some films did promote diets containing a cornucopia of fibre, beneficial proteins or polyphenols. These are the gifts under the Christmas tree for our microbiota.

**Conclusions:**

Good balance is needed in our microbiota, and consequently influences our mental health. Many festive films portray a “Western diet”, which leads to dysbiosis. Through the gut-brain axis and the influence of media, the festive foods eaten in these films (maybe an extra chocolate biscuit during Love Actually) may cause stress to our microbiota.

**Disclosure of Interest:**

None Declared

